# Research on the Properties of Polysaccharides, Starch, Protein, Pectin, and Fibre in Food Processing

**DOI:** 10.3390/foods12020249

**Published:** 2023-01-05

**Authors:** Xin Qi, Yanjun Zhang, Hansong Yu, Jianhua Xie

**Affiliations:** 1 State Key Laboratory of Food Science and Technology, Nanchang University, Nanchang 330047, China; 2 Spice and Beverage Research Institute, Chinese Academy of Tropical Agricultural Sciences, Wanning 571533, China; 3 College of Food Science and Engineering, Jilin Agricultural University, Changchun 130118, China

As food components, polysaccharides, starch, protein, pectin, and fibre are often used in the food industry due to their particular functional properties, as well as their efficient, safe, and green characteristics. As shown in Table 1, they have been used to improve food texture, increase freshness and shelf-life, produce food packaging materials and health care products, deliver food active ingredients, and so on. However, these native components still have some drawbacks, such as lower solubility and instability under certain conditions, which limit their application in food processing. Therefore, the modification of natural food components by physical, chemical, and enzymatic means seeks to obtain components with superior functional properties and to expand their applications in the food field. In this sense, a Special Issue entitled “Research on the Properties of Polysaccharides, Starch, Protein, Pectin, and Fibre in Food Processing” was launched in *Foods* (MDPI) to provide a forum for researchers to communicate their latest research findings on polysaccharides, starch, protein, and other food components. A total of 63 manuscripts were submitted to this Special Issue from various countries, of which 34 were accepted for publication in the peer-review process, including 4 reviews and 30 research articles.

Among these published papers, there were plenty of studies on polysaccharide biological activities. Li et al. [12] isolated and purified polysaccharides from yam. They found the molecular weight of yam polysaccharides was 20.89 kDa, which primarily comprised galactose, glucose, and galacturonic acid with a ratio of 28.57:11.28:37.59. Moreover, pretreatment with yam polysaccharides attenuated oxidative damage in IEC-6 cells by H_2_O_2_ through the mitogen-activated protein kinase (MAPK) signalling pathway, improved cell viability, superoxide dismutase (SOD) activity, and reduced intracellular reactive oxygen species (ROS) production and malondialdehyde (MDA) content after H_2_O_2_ injury. Therefore, yam polysaccharides possess a good protective effect against oxidative damage, which could provide a reference for the development of functional foods and clinical therapeutics against the damage resulting from oxidative stress. Wang et al. [13] revealed that *Ganoderma atrum* (*G. atrum*) polysaccharide (PSG-1) activated the mammalian target of the rapamycin (mTOR) signalling pathway, and also alleviated the acrolein-induced impairment of viability, autophagy, and apoptosis of IEC-6 cells. Moreover, another study reported that short-chain fatty acids in the faeces of mice treated with high *Holothuria leucospilota* polysaccharides (HLP) doses were significantly higher than those treated with lower doses and the normal group. After oral administration of HLP, the glutathione peroxidase and SOD activities increased, and malondialdehyde contents in the mouse livers decreased, which revealed the good performance of HLP with respect to liver antioxidants [14]. Natural polysaccharides come from a wide range of sources; in addition to higher plants, microorganisms (bacteria and fungi) and algae are also rich in polysaccharides. Sun et al. [15] found that a polysaccharide produced by *Chaetomium globosum* CGMCC 6882 had antibacterial activity, and it enriched the abundance of gut microbiota and the firmicutes/bacteroidetes ratio was increased from 0.52 to 0.72. Chemical selenylation enhanced the immuno-modulatory effect of *Portulaca oleracea* L. polysaccharides (PSPO) on the murine splenocytes and RAW 264.7 macrophages [16]. In recent years, various natural polysaccharides, including *Dictyophora indusiata* polysaccharides, *Sarcodon aspratus* polysaccharides, *Flammuliana velutipes* polysaccharides, and *Pleurotus eryngii* polysaccharides, have shown great potential in attenuating systemic inflammation activities, such as inflammatory bowel disease (IBD). Wang et al. [17] summarized the main mechanisms of polysaccharides on IBD, including immune regulation, anti-oxidation, and regulation of probiotics in the intestine. Moreover, some polysaccharides were found to help control type 2 diabetes with minimal side effects. Wan et al. [18] reported that potential mechanisms for the antidiabetic function of β-glucan may include the retardation of macronutrient absorption and the inhibition of digestive enzymes. In addition to having a variety of biological activities, polysaccharides are also a good delivery carrier. Ferron et al. [19] used a polysaccharide fraction isolated from *Camelina sativa* L. Krantz (CCP) as a carrier for purple corn cob extract (MCE), and they found CCP could supply a protective barrier for MCE, effectively increasing storage stability and bioaccessibility. At the same time, polysaccharide-based materials have advantages such as edibility and environmentally friendly performance; as such, polysaccharides as a new kind of packaging material have been proposed. The most commonly used polysaccharide as a packaging material is chitosan. Chitosan has good biological activities, including antibacterial and antioxidant activity and enzyme activity inhibition, and thus possesses great potential in the field of food packaging and preservation [20]. Additionally, cellulose, hemicellulose and starch are also important natural sources of packaging materials. Liu et al. [20] and Zhao et al. [21] reviewed the latest advances in polysaccharide-based materials in food packaging and provided references for the development of modern edible packaging and novel preservation technologies.

Starch is widely used in the food industry, primarily in bakery products, noodles, instant foods, and snacks. The physicochemical properties of starches, such as pasting, rheological and retrogradation behaviours, could determine the qualities of their end products. However, many native starches have some drawbacks, such as poor solubility, stability against heat and shear during pasting, undesired paste consistency, and easy retrogradation. These drawbacks have limited the application of starch in the food industry. Therefore, many modification methods, such as chemical, enzymatic, and physical methods, or some combination of these, have been used to enhance the physicochemical properties of native starch to meet the demands of consumers. Xu et al. [22] modified potato starch by a pre-gelatinization method and built correlations between starch physicochemical properties and the degree of starch gelatinization (DSG). They found that the endothermic enthalpy, gelatinization range, and short-range ordered structure of starch were negatively correlated with DSG, whereas onset gelatinization temperature, apparent viscosity, and water-binding capacity were positively correlated. The viscoelasticity of starch gels was negatively correlated with the DSG after full gelatinization (DSG > 39.41%). Starch granules gradually lost their typical shape, and less birefringence could be observed with increasing DSG. Dai et al. [23] adjusted the physicochemical properties of rice, potato, and pea starches with betanin. Betanin decreased the peak, trough, and final viscosity of rice and potato starches, but increased those of pea starch. The poor short-range molecular order, low crystallinity, and low retrogradation enthalpy of starches were induced by betanin during retrogradation, suggesting that betanin could inhibit the retrogradation of starches. Similarly, Xu et al. [24] investigated the effect of different degrees of polymerization (DP) of polymeric proanthocyanidins (PPC) on the physicochemical characteristics and in vitro starch digestibility of potato starch. PPC enhanced the thermal stability, delayed starch gelatinization of potato starch, and affected the elastic properties, instead of the viscous properties, of potato starch pasting. PPC with a lower DP more evidently influenced starch gelling performance, whereas larger PPC molecules exhibited a greater impact on starch long-term retrogradation and digestive properties. In another study, native starch and fermented starch featured significantly higher hygroscopicity than starch granulate [25]. Resistant starch is the sum of starch and starch degradation products not absorbed by the small intestine of a healthy individual, which could improve insulin resistance and glucose homeostasis, maintain colon health, control body weight, elevate large-bowel short-chain fatty acids, and especially lower blood lipid. Zhang et al. [26] evaluated the effects of jackfruit seed sourced resistant starch (JSRS) on mice gut microbes and hyperlipidaemia. JSRS promoted the growth of *Bifidobacterium pseudolongum*, and *Bifidobacterium pseudolongum* interacted with JSRS to significantly reduce bodyweight and serum lipid levels, having a therapeutic effect on hepatic steatosis in mice. Their findings will help in the development of “synbiotics” for the treatment of hyperlipidaemia.

Dietary fibre is a polysaccharide that can neither be digested or absorbed by the gastrointestinal tract, and it is recognized as a seventh category nutrient by the nutritional supplement community. Therefore, improving the quality and functional properties of insoluble dietary fibre through physical, chemical, and biological approaches is a prerequisite for its wide application in the food sector. He et al. [27] used a microfluidizer system to improve the structures and functionalities of pea fibre. Cheng et al. [28] investigated the effects of γ-irradiation on the structure and functional properties of pea fibre. Their results showed that, when the γ-irradiation dose was 2 kGy, the highest oil-holding capacity, swelling capacity and water-holding capacity of pea fibre were 8.12 ± 0.12 g/g, 19.75 ± 0.37 mL/g, and 8.35 ± 0.18 g/g, respectively. γ-irradiation technology as an effective method significantly improved the functional properties of pea fibre. Lyu et al. [29] explored the properties of high-purity insoluble dietary fibre from *Okara* (HPIDF); after fermentation, it showed a higher water-holding capacity, water-swelling capacity, heavy-metals-adsorption capacity, and harmful-substances-adsorption capacity than HPIDF due to the changes in structure caused by fermentation. Based on the above studies, it is clear that dietary fibre changed its own properties after fermentation. Does the presence of dietary fibre affect the fermentation of other substances in the intestine? Yang et al. [30] explored the effect of four dietary fibres (fructose-oligosaccharides, pectin, β-glucan and arabinoxylan) on the modulation of cyanidin-3-O-glucoside (C3G) fermentation patterns. The results showed that the C3G metabolites after in vitro fermentation mainly included cyanidin, protocatechuic acid, 2,4,6-trihydroxybenzoic acid, and 2,4,6-trihydroxybenzaldehyde. Dietary fibres changed the proportions of C3G metabolites, which were dependent on the dietary fibres structures. It is well known that dietary fibre is an important component for promoting human health and managing calorie consumption. Fan et al. [31] showed that the *Okara* insoluble dietary fibre of three particle sizes could improve the elevation of blood lipids and the disturbance of gut microbiota caused by a high-fat diet; this finding provides valuable information regarding its future application in functional foods. In addition, dietary fibre is also a good carrier for the protection of bioactive substances. He et al. [32] found that soybean insoluble dietary fibre effectively improved the stability of malvidin-3-O-glucoside under different pH values (1.0~7.0), high temperatures, and sunlight conditions. 

Protein is widely used in all kinds of foods, such as candy, pastry, dairy products, and so on. It not only enhances nutrition but can improve the quality and texture of various foods due to gelation, water holding and binding capacities, foaming ability, and stability, solubility, emulsification, etc. However, the functional properties of many proteins do not meet expectations, so multiple methods (e.g., physical, chemical, and enzymatic modifications) have been used to improve the functional properties of proteins. Pang et al. [33] prepared (−)-epigallocatechin gallate (EGCG)-hemp (*Cannabis sativa* L.) seed protein (HPI) conjugates to modify HPI. The solubility and emulsifying properties of the EGCG-HPI conjugates were improved compared with native HPI. Enzymatic hydrolysis was considered as a promising method for the improvement in functional properties of protein. Shuai et al. [34] found enzymatic modification treatment distinctly boosted the solubility of pea protein (PP), while the foaming and emulsification of PP were improved after enzymatic treatment. Zhang et al. [35] reported that the foaming property and water/oil holding capacity of walnut protein isolates were improved after oxidation by 2,2′-azobis (2-amidinopropane) dihydrochloride. Isoelectric solubilization/precipitation (co-precipitation) was used to produce protein mixtures from pea (PPI) and grass carp (CPI) [36]. The solubility and emulsifying properties of co-precipitates (PPI-CPI) were greater than those of PPI, CPI, and isolated protein blends (BL), and the foaming activity was higher than that of BL and CPI. Zhou et al. [37] mixed these four protein systems (PPI-CPI, PPI, CPI, BL) with soybean oil and water to form a pre-emulsification system to replace animal fat and prepare fish sausage. Their results showed that the overall quality of PPI-CPI fish sausage, as a whole, was significantly superior to that of PPI and BL, which was equivalent to CPI fish sausage. PPI-CPI fish sausage had a significantly higher water-holding capacity and hardness (*p* < 0.05). Generally, protein has gelling properties, and protein-based gelled products have become an increasingly popular food, such as surimi-based products. Heat treatment and the addition of some exogenous substances determine the quality of the gel matrix. Jiang et al. [38] reported that starch addition had a positive effect on the weak gel matrix by direct heating. Zhu et al. [39] found that carboxymethyl chitosan oligosaccharide stabilized the microstructure of the surimi gels associated with improved gel strength, viscoelasticity, water-holding capacities, and whiteness during frozen storage. Their studies are a route by which to widen the development of protein-based gelled products, and they provide useful information for food cryoprotectants. From the above studies, it is clear that proteins and polysaccharides are often added to the same system to better nullify the disadvantages of protein and polysaccharide alone, such as poor stability, gelling properties, etc. Li et al. [40] prepared zein nanoparticles/flaxseed gum complexes to stabilize Pickering emulsion. The emulsions remained stable without a serum phase for 14 days and exhibited improved stability at low-temperature storage; this provides guidance for the preparation of protein–polysaccharide complexes and Pickering emulsions. In the complex food system, food constituents (pectin, starch, sucrose, and protein) interact with each other and thus change their properties. Liu et al. [41] showed food constituents in the food system decreased the activity of polyphenol oxidase but increased the thermostability of polyphenol oxidase, and pectin exhibited the strongest protective effect against thermal inactivation. Therefore, it is meaningful to study the interactions between food macromolecules because they could affect molecular properties and food texture. Zhang et al. [42] studied the interaction between gelatin and *Mulberry* leaf polysaccharides obtained by four different extraction conditions (hot buffer = HBSS; chelating agent = CHSS; dilute alkali = DASS; concentrated alkali = CASS). They found that the addition of gelatin reduced the apparent viscosity of the four polysaccharide solutions. Gelatin-polysaccharides obtained by CHSS were highly stable, and gelatin-polysaccharides obtained by CASS were more suitable as stabilizers in the freezing process. Therefore, different extraction methods affect the properties of polysaccharides. He et al. [43] optimized the extraction process of quinoa (*Chenopodium quinoa* Willd.) protein isolate (QPI), and found the hydrolysis degree and total amino acid content at the end of digestion were significantly lower than those untreated at 90 °C and 121 °C for 30 min. Therefore, heat treatment could affect the vitro digestion characteristics of QPI. Among the many food proteins, there are some proteins, such as egg protein and peanut protein, which can cause allergic reactions and give consumers an unpleasant experience. Therefore, various studies have focused on exploring effective and safe technologies that might reduce the allergenicity of proteins. Wen et al. [44] used an emerging and efficient technology, superheated steam (SS), to change the allergenicity and molecular structure of ovomucoid (OVM). The IgG-binding ability of OVM decreased and the releases of β-hexosaminidase and TNF-γ were inhibited after SS treatment. The natural OVM structure was disrupted, leading to increased protein digestibility. Moreover, peptide Phe-Thr-Gly-Met-Leu (FTGML) inhibited the tyrosinase activity and melanin synthesis of B16F10 melanoma cells. FTGML reduced melanogenesis in B16F10 cells by downregulating the cAMP-PI3K/Akt and MAPK pathways (p38 and JNK) [45].

In short, the articles in this Special Issue cover various fields such as improvements in the physical properties of major food components, biological activity studies, and applications in food preservation. The pursuit of food texture and taste and the stringent requirements for safe, green, and efficient food materials are the main driving forces for continued research in this field. 

## Figures and Tables

**Table 1:** The properties and applications of food components in food.

Food Components	Properties	Applications in Food	Refs.
Polysaccharides	Antibacteria, immunomodulatory, anti-tumour, hypoglycaemic, hypolipidemic, anti-oxidation effects, thickening, stabilizing, gelling and emulsifying properties, etc.	Packaging materials, preservative, health care products, food active ingredient carriers, texture modifiers, etc.	[1,2,3]
Starch	Thickening, stabilizing, gelling, emulsifying, film-forming properties, water holding capacity, etc.	Ingredient of gelled foodstuff, texture modifiers, biodegradable packaging materials, food active ingredient carriers, etc.	[4,5]
Protein	Gelation, water holding and binding capacities, foaming ability and stability, solubility, emulsification, etc.	Texture modifiers, nutrient supplements, plant-based meats, fat replacement, food active ingredient carriers, edible packaging film, etc.	[6,7]
Pectin	Thickening, stabilizing, gelling, emulsifying, film-forming properties, water and oil holding capacities, reduce blood lipid, cholesterol, radiation resistance, adsorption of heavy metal ions capacities, etc.	Texture modifiers, edible packaging film, antioxidant, fat replacement, health care products, food active ingredient carriers, etc.	[8,9]
Fibre	Water holding, water swelling, water retention, oil holding, glucose retardation index, stabilizing, gel-forming, antioxidant capacities, water solubility, etc.	Texture modifiers, antioxidant, fat replacement, product freshness and shelf-life fortifier, food packaging, etc.	[10,11]
